# Stage-dependent tau-PET signatures in Huntington’s disease revealed by [¹⁸F]PI-2620

**DOI:** 10.1007/s00259-026-08003-0

**Published:** 2026-06-29

**Authors:** Saul Martinez-Horta, Jesus Perez-Perez, Arnau Puig-Davi, Valle Camacho, Laura Perez-Carasol, Carla Franch-Marti, Gonzalo Olmedo-Saura, Alex Fernandez-Leon, Albert Flotats, Guilherme D. Kolinger, Santiago Bullich, Esther Perez-Martinez, Javier Pagonabarraga, Jaime Kulisevsky

**Affiliations:** 1 Movement Disorders Unit, Neurology Department, Hospital de la Santa Creu i Sant Pau, Barcelona, 08041 Spain; 2 Research Institute Sant Pau (IR-Sant Pau), Centre CERCA, Barcelona, Spain; 3 Centro de Investigación Biomédica en Red-Enfermedades Neurodegenerativas (CIBERNED), Madrid, Spain; 4 Department of Medicine, Autonomous University of Barcelona, Barcelona, Spain; 5 European Huntington’s Disease Network (EHDN), Ulm, Germany; 6 Nuclear Medicine Department, Hospital de la Santa Creu i Sant Pau, Barcelona, Spain; 7 Life Molecular Imaging GmbH, Berlin, Germany; 8 Lantheus Switzerland GmbH, Basel, Switzerland

**Keywords:** Huntington’s disease, Tau PET, [¹⁸F]PI-2620, Disease stage, Kinetic modelling, Neurodegeneration

## Abstract

**Purpose:**

Neuropathological and biomarker evidence implicates tau dysregulation as a downstream component of Huntington’s disease (HD) pathobiology, yet its in vivo distribution has not been characterised using second-generation tau-PET tracers. We aimed to define the regional organisation, stage dependence and clinical relevance of tau-sensitive PET signal across the HD disease spectrum.

**Methods:**

Fifty-four participants (13 healthy controls, 9 premanifest mutation carriers and 32 manifest carriers) underwent 60-minute dynamic [¹⁸F]PI-2620 PET imaging. Tau-PET signal was quantified using distribution volume ratios (DVR) derived from reference-tissue kinetic modelling. Analyses combined region-of-interest and whole-brain mapping with threshold-based positivity profiling, modelling of cumulative genetic disease burden (CAP), and clinico-anatomical association analyses.

**Results:**

Tau-PET abnormalities showed a spatially ordered pattern dominated by subcortical involvement. The globus pallidus exhibited the strongest effect, with marked DVR increases and high positivity rates emerging in premanifest carriers and approaching saturation in manifest HD. Additional subcortical changes involved the putamen, whereas caudate DVR decreased in manifest disease. Cortical effects were more modest and selectively involved posterior associative regions. CAP modelling supported predominantly monotonic subcortical stage-related profiles, while limbic and cortical regions showed heterogeneous cross-sectional patterns. Clinico-anatomical analyses linked subcortical tau-PET signal with genetic burden and motor severity and limbic signal with psychiatric symptoms.

**Conclusion:**

Tau-sensitive PET signal represents a spatially ordered and stage-dependent feature of HD and identifies a reproducible pallidal signature linked to disease burden, supporting its potential as a biomarker for patient stratification and mechanistic monitoring in therapeutic studies targeting downstream pathological processes.

**Trial registration number:**

NCT07503743.

**Supplementary Information:**

The online version contains supplementary material available at https://doi.org/10.1007/s00259-026-08003-0.

## Introduction

Huntington’s disease (HD) is a fully penetrant monogenic neurodegenerative disorder caused by an abnormal expansion of CAG repeats in the *HTT* gene, which initiates a cascade of molecular and cellular abnormalities leading to progressive neuronal dysfunction and loss [1, 2]. Despite this genetic determinism, HD displays striking clinical heterogeneity in age at onset, cognitive trajectories, motor phenotype, and rate of neurodegeneration [2–8]. This variability, frequently observed even among individuals with comparable CAG lengths, indicates that clinical expression of HD is not determined by CAG mechanisms alone. Within this framework, multiple downstream processes beyond CAG repeat length are thought to contribute to HD heterogeneity, including somatic expansion, transcriptional dysregulation, synaptic dysfunction, neuroinflammation, and impaired protein homeostasis [2, 3, 9–18].

Among these processes, increasing evidence implicates tau pathology as a recurring but incompletely understood feature of HD [19–21]. Historically, tau pathology in HD has often been interpreted as a secondary consequence of neurodegeneration or attributed to concomitant Alzheimer-type pathology, primary age-related tauopathy (PART), aging-related tau astrogliopathy (ARTAG), or other age-related copathological processes [22–28].

Human neuropathological evidence for tau involvement in HD is heterogeneous and should not be reduced to a single model of classical Alzheimer-type neurofibrillary pathology. Early studies reported Alzheimer-type tau lesions in subsets of HD brains, including Braak stages I–III in approximately 60% of cases [29], while severe Alzheimer-type copathology has been described in elderly HD patients with prominent dementia [30]. More recent neuropathological work has also shown tau copathology in HD limbic regions, with Braak tau stages I–III observed across several HD brains and Vonsattel stages, together with other proteinopathies such as amyloid-β and TDP43 [31]. Conversely, Baskota et al. emphasized that tau lesions in HD may often overlap with PART, ARTAG, low-level Alzheimer-type neuropathological change or other secondary age-related entities [26]. Thus, HD should not be framed as a uniform primary tauopathy, but the available human literature also argues against considering tau involvement absent or biologically irrelevant.

Beyond Braak-type lesions, human post-mortem and mechanistic studies support a disease-linked disturbance of tau biology in HD. HD brains show altered tau homeostasis, including increased total tau, 4R predominance, higher 4R/3R ratio, altered MAPT splicing, and nuclear/cytoskeletal tau abnormalities linked to mHTT-related disturbances in RNA processing and microtubule regulation [19, 20, 32–34]. Additional studies have reported hyperphosphorylated tau aggregates, oligomeric tau species, tau pathology within neural grafts exposed to the HD host environment, altered tau phosphorylation and subcellular distribution induced by mutant huntingtin, soluble truncated tau species related to cognitive dysfunction, and tau hyperphosphorylation linked to phosphatase dysregulation [19, 20, 32, 35, 36]. Mechanistically, these findings converge on pathways involving MAPT alternative splicing, increased exon 10 inclusion and 4R tau predominance, region-specific regulation of tau exons 2 and 10 in vulnerable regions such as the putamen [37], phosphatase dysfunction, altered tau localization, and generation of oligomeric or soluble tau species. These abnormalities do not require abundant mature neurofibrillary tangles to be biologically meaningful, and support tau dysregulation as a variably expressed downstream component of HD pathobiology. In vivo studies of plasma and CSF tau biomarkers remain heterogeneous [38–41], but some have linked tau measures to posterior cortical neurodegeneration, disease burden and cognitive performance [23, 42].

Despite this convergent evidence, tau involvement in HD has not been characterised in vivo using second-generation tau-PET tracers [43–45]. Consequently, it remains unclear whether tau-sensitive abnormalities emerge early in the disease course, follow defined anatomical patterns, or primarily reflect individual differences in genetic burden, neurodegeneration, tracer kinetics or copathological processes. This distinction is particularly important in HD, where tau abnormalities may be low-level, heterogeneous, non-classical and regionally dependent, and where PET signal should not be equated directly with classical fibrillar tau burden.

Second-generation tau-PET enables regionally specific and stage-sensitive in vivo assessment of tau-sensitive signal. [¹⁸F]PI-2620 is a second-generation tau PET tracer with extensive preclinical, autoradiographic and in vivo validation. Preclinical studies demonstrated high-affinity binding to tau deposits, favourable selectivity over amyloid-β, low non-specific white-matter binding, and binding to both mixed 3R/4R and 4R tau preparations, including recombinant 4R tau fibrils and PSP brain homogenates [46]. In vivo studies in Alzheimer’s disease and primary 4R tauopathies have further supported its ability to detect tau-related signal [43–45, 47, 48], including autoradiographic and in vivo evidence of binding in PSP target regions such as the globus pallidus, subthalamic nucleus and midbrain [49]. Additional studies suggest that [¹⁸F]PI-2620 binding characteristics may differ across 3R/4R and 4R tauopathy phenotypes [50], while post-mortem radioligand studies support high-affinity PI-2620 binding in Alzheimer’s disease and some non-Alzheimer tauopathy tissues, although binding strength and specificity in 4R tauopathies remain debated [51, 52]. Thus, [¹⁸F]PI-2620 should be regarded as a tau-sensitive tracer with strong tau-binding rationale, while recognizing that tracer retention in non-Alzheimer conditions may depend on tau conformation, acquisition window, tissue properties, disease stage and kinetic behaviour.

Within this framework, distribution volume ratio (DVR) should be interpreted as an operational kinetic outcome derived from reference-tissue modelling rather than as a direct one-to-one measure of fibrillar tau burden. In low-density or non-classical tau contexts such as HD, DVR differences may reflect several non-mutually exclusive components, including low-level or non-classical tau binding, binding to tau isoforms or conformations distinct from Alzheimer-type neurofibrillary tangles, altered tissue kinetics, loss of tracer-accessible substrate in neurodegenerated tissue, partial-volume effects, reference-region variability and potential non-specific or off-target components. The use of fully dynamic [¹⁸F]PI-2620 PET and reference-tissue kinetic modelling provides a physiologically grounded approach to characterising tracer behaviour, but the molecular substrate of [¹⁸F]PI-2620 retention in HD remains to be established by HD-specific autoradiographic and post-mortem validation studies.

Accordingly, the present study uses dynamic [¹⁸F]PI-2620 PET imaging across the clinical spectrum of HD to (i) map the regional and cross-sectional stage-related distribution of tau-sensitive PET signal, (ii) examine relationships with clinical and psychiatric heterogeneity, and (iii) assess the influence of cumulative genetic disease burden on in vivo [¹⁸F]PI-2620 PET abnormalities. Throughout the manuscript, we refer to the measured signal as tau-sensitive rather than tau-specific, reflecting both the strong tau-binding rationale of the tracer and the current absence of HD-specific post-mortem binding validation.

## Materials and methods

### Participants

A total of 54 individuals were included: 13 healthy controls, 9 premanifest mutation carriers, and 32 manifest carriers classified according to HD-ISS [53]. Genetic variables included CAG repeat length and the CAG–Age Product (CAP) score [54]. Written informed consent was obtained from all participants. The study was approved by the local ethics committee and conducted in compliance with the Declaration of Helsinki and its later amendments.

### Clinical assessments

Global disease severity was assessed with the composite Unified Huntington’s Disease Rating Scale (cUHDRS) [55]. Motor impairment and functional capacity were evaluated using the UHDRS total motor score (UHDRS-TMS) and Total Functional Capacity (TFC) scale, respectively [56, 57]. Cognitive status was assessed with the Parkinson’s Disease–Cognitive Rating Scale (PD-CRS) [58–60], and neuropsychiatric symptoms with the short form of the Problem Behaviors Assessment (PBA-s) [61, 62]. Additional rationale for the selection of the PD-CRS is provided in the Supplementary Methods (S1).

### MRI acquisition and processing

All participants underwent high-resolution structural MRI on a 3 Tesla Philips Ingenia scanner using a T1-weighted MP-RAGE sequence. Structural MRI was used for PET co-registration, spatial normalisation, and tissue segmentation. PET images were registered to the individual T1 image and nonlinearly normalised to MNI space. Gray matter probability maps were intersected with the Automated Anatomical Labeling (AAL) atlas to define gray-matter–constrained regions of interest, reducing cerebrospinal fluid contamination and atrophy-related spill-out effects [63]. Full MRI acquisition parameters are provided in the Supplementary Methods (S2).

### PET acquisition and quantification

[¹⁸F]PI-2620 PET imaging was performed on a Philips Vereos scanner (Eindhoven, The Netherlands). Dynamic acquisition started at tracer injection and continued for 60 min. After visual quality control, dynamic frames were motion-corrected, co-registered to each participant’s T1-weighted MRI, and spatially normalised as described above. Point-spread-function modelling was not enabled during image reconstruction, and no post-reconstruction partial-volume correction was applied.

Tracer binding was quantified using distribution volume ratios (DVR) derived from the multilinear reference tissue model (MRTM2), a validated approach for dynamic [¹⁸F]PI-2620 imaging [55]. Cerebellar grey matter (excluding vermis and adjacent anterior lobe) was used as the reference region. DVR estimates constituted the primary outcome measure for all subsequent analyses and was interpreted as an operational reference-tissue kinetic outcome reflecting relative tracer distribution between target and reference tissue. Full acquisition, reconstruction, and modelling parameters are provided in the Supplementary Methods (S2).

### Statistical analysis

#### Clinical and demographic characterization

Descriptive analyses were first undertaken to characterise the demographic, genetic, and clinical profile of the sample across three groups. Continuous variables were summarised using means and standard deviations, and categorical variables using frequencies and percentages. Group differences in demographic and clinical measures were evaluated with general linear models or ANCOVA, adjusting for age and sex when appropriate. For ordinal or non-normally distributed outcomes, non-parametric tests were used. For continuous outcomes with more than two groups, post-hoc pairwise comparisons were derived from the fitted models, with adjusted means and 95% confidence intervals. Effect sizes were calculated to quantify the magnitude of between-group differences and to contextualise subsequent imaging findings. Additional statistical details are provided in the Supplementary Methods (S3.1).

#### PET analysis framework

Inferential analyses were conducted primarily at the region-of-interest (ROI) level using PET-derived DVR measures. ROIs were defined according to the Automated Anatomical Labeling (AAL) atlas and subsequently aggregated into anatomically coherent macro-regions [63]. The analytical hierarchy was defined a priori to distinguish primary from complementary analyses. Primary analyses focused on group differences in ROI-based DVR measures, with particular emphasis on subcortical regions and the globus pallidus given the observed effect-size hierarchy and biological relevance. For descriptive spatial mapping, complementary analyses were also performed across the full set of AAL regions to generate cortical and subcortical brain maps. Group differences in tau-sensitive PET signal were evaluated using general linear models with HD-ISS group as the main factor and age and sex included as covariates. For primary ROI-based analyses, false discovery rate (FDR) correction was applied using the Benjamini–Hochberg procedure (q = 0.05). Positivity profiling, full AAL mapping, quadratic CAP models, clinical × CAP interaction models and ROC analyses were considered complementary or exploratory analyses intended to characterize regional heterogeneity and generate hypotheses for future studies. Additional details regarding the regional framework and multiregional interpretation are provided in the Supplementary Methods (S3).

To characterise the regional emergence of tau-PET abnormalities, ROI-based positivity analyses were performed using DVR-derived z-scores calculated relative to the healthy control group. Positivity was defined a priori as z ≥ 1.5, and the proportion of positive cases was quantified for each region and disease stage. Complete region-wise results are provided in the Supplementary Material (Supplementary Table 2), with additional methodological details reported in the Supplementary Methods (S4). CAP-dependent effects on regional positivity were evaluated in mutation carriers using age- and sex-adjusted logistic regression models including linear and exploratory quadratic CAP terms (Supplementary Methods S5).

Associations between regional tau-PET signal and clinical measures were examined in mutation carriers using age- and sex-adjusted linear regression models based on DVR values extracted from anatomically aggregated macro-regions. Where exploratory analyses suggested heterogeneous patterns, additional interaction models incorporating clinical measure × CAP terms and quadratic CAP effects were evaluated. Regression coefficients are reported as standardised β values, and FDR correction (q = 0.05) was applied across regions within each clinical measure. Full model specifications are provided in the Supplementary Methods (S6).

The individual-level discriminative value of tau-PET was assessed using receiver operating characteristic (ROC) analyses based on regional DVR values. Area under the curve (AUC) estimates and optimal thresholds were calculated using Youden’s J statistic, with confidence intervals obtained by non-parametric bootstrapping (1,000 resamples). Statistical significance was evaluated using DeLong’s test.

## Results

### Clinical and sociodemographic characteristics

As shown in Table 1, groups did not differ significantly in age, sex distribution, or years of education. Cumulative genetic disease burden increased markedly across stages, with higher CAP scores in manifest compared with premanifest carriers [F(1,39) = 16.91, *p* = 0.0002]. Clinical measures showed the expected stage-related deterioration. Global disease severity (cUHDRS) remained comparable between HC and preHD but declined substantially in manifest HD [F(2,51) = 34.72, *p* < 0.0001]. Motor impairment increased across groups (UHDRS-TMS: F(2,51) = 32.53, *p* < 0.0001), accompanied by reduced functional capacity (TFC: F(2,51) = 13.77, *p* < 0.0001). Cognitive performance (PD-CRS) was preserved in HC and preHD but markedly reduced in manifest HD [F(2,51) = 24.73, *p* < 0.0001]. Among neuropsychiatric measures, apathy and perseveration showed the clearest stage-related increases (apathy: F(2,51) = 10.29, *p* = 0.0002; perseveration: F(2,51) = 16.84, *p* < 0.0001).

**Table 1:** Demographic, Clinical, Cognitive and Neuropsychiatric Characteristics of the sample

Variable	HC	preHD	HD	*p*-value	Cohen’s d
Age (years)	51.77 (12.66)	46.22 (11.76)	53.16 (11.16)	0.1250	−0.13
Sex (% male)	23.1%	22.2%	50.0%	0.130 (χ²)	-
Education (years)	13.92 (4.21)	14.67 (4.24)	13.28 (3.53)	0.6100	0.16
CAG	-	42.11 (2.62)	44.06 (3.78)	0.1600	-
CAP score	-	375.60 (90.67)	518.98 (92.85)	0.0002	-
cUHDRS	17.80 (2.06)	17.26 (1.78)	9.21 (4.38)	< 0.0001	2.68
UHDRS-TMS	0.00 (0.00)	2.33 (2.35)	36.66 (20.48)	< 0.0001	−2.11
TFC	13.00 (0.00)	13.00 (0.00)	10.28 (2.40)	0.0000	1.33
PBAs					
*Depression*	0.62 (1.50)	0.44 (1.33)	1.03 (1.80)	0.5640	−0.24
*Suicidality*	0.00 (0.00)	0.00 (0.00)	0.12 (0.49)	0.5050	−0.30
*Anxiety*	2.00 (2.27)	1.44 (1.88)	0.84 (1.74)	0.1790	0.61
*Irritability*	0.62 (1.19)	0.56 (1.33)	1.09 (1.86)	0.4210	−0.33
*Aggression*	0.15 (0.38)	0.44 (1.13)	1.06 (1.65)	0.2570	−0.42
*Apathy*	0.00 (0.00)	1.00 (2.00)	2.94 (2.54)	0.0002	−1.40
*Perseveration*	0.00 (0.00)	0.44 (1.33)	3.10 (2.21)	0.0000	−1.63
*OCD*	0.15 (0.55)	0.22 (0.83)	0.56 (1.43)	0.3680	−0.31
*Delusions*	0.00 (0.00)	0.00 (0.00)	0.06 (0.25)	-	-
*Hallucinations*	0.00 (0.00)	0.00 (0.00)	0.03 (0.18)	0.7170	−0.21
*Disorientation*	0.00 (0.00)	0.00 (0.00)	0.03 (0.18)	0.7170	−0.21
PD-CRS Total	108.85 (14.68)	108.11 (13.71)	76.90 (17.19)	< 0.0001	1.91
*PD-CRS Subcortical*	79.85 (13.22)	79.22 (13.01)	50.34 (15.33)	< 0.0001	2.00
*PD-CRS Cortical*	28.54 (1.56)	28.89 (0.78)	26.56 (3.40)	0.0277	0.66

*CAG* CAG repeat length, *CAP* *score* CAG x AGE product, *cUHDRS* Composite UHDRS, *UHDRS*-*TMS* Unified Huntington’s Disease Rating Scale Total Motor Score, *TFC* Total Functional Capacity, *PBAs* Problems Behavior Assessment Scale (Short form). Data is expressed as mean (SD) for continuous variables and as percentages for categorical variables. Cohen’s d reflects effect sizes for pairwise comparisons between control and manifest HD groups

### Tau-sensitive PET signal patterns assessed with dynamic DVR

#### Group differences in dynamic DVR

**Fig. 1:**
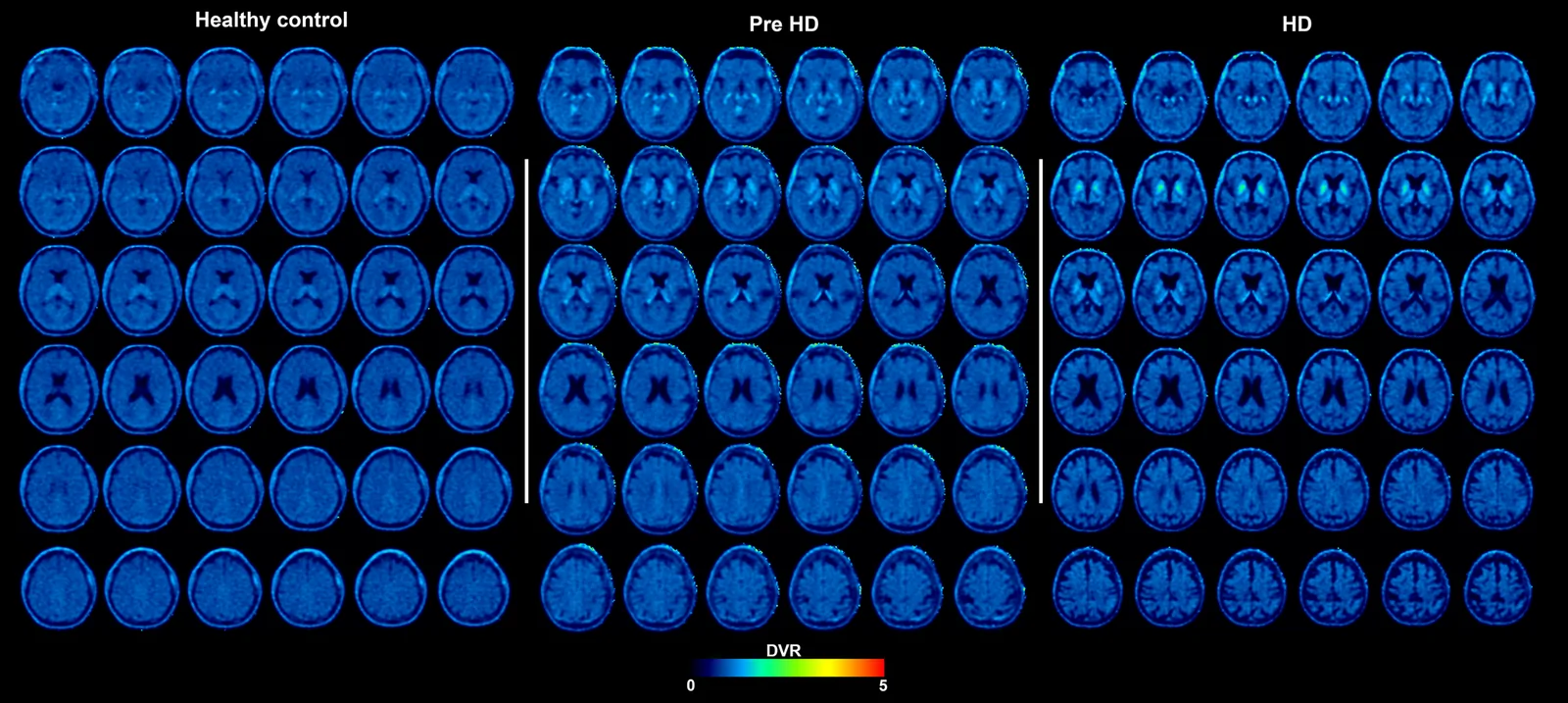
Representative individual-level tau-sensitive PET signal patterns across disease stages (DVR). Representative axial slices showing individual-level [¹⁸F]PI-2620 PET signal quantified using DVR in a healthy control, a premanifest mutation carrier, and a participant with manifest HD. Images are displayed using a common anatomical orientation and colour scale. The examples illustrate minimal and homogeneous DVR signal in the control participant, focal and regionally selective subcortical DVR elevations in premanifest disease, and more pronounced and consolidated subcortical and limbic DVR signal in manifest HD. Images are shown for qualitative illustration and do not represent group averages

Visual inspection of representative DVR maps revealed clear cross-sectional stage-related differences in [¹⁸F]PI-2620 signal (Fig. 1). Healthy controls showed low and spatially homogeneous tracer uptake. Premanifest carriers exhibited focal DVR increases predominantly in the globus pallidus and adjacent basal ganglia structures, with minimal cortical involvement. In manifest HD, subcortical binding intensified and extended to additional deep nuclei and brainstem regions, while cortical signal remained modest and anatomically structured, mainly involving posterior associative cortices.

At the group level, tau-sensitive PET signal quantified using dynamic DVR showed a highly structured pattern of disease-related differences across the HD spectrum. Subcortical regions showed the most prominent abnormalities. The globus pallidus exhibited the largest effect in the entire brain, with mean DVR increasing from 0.97 ± 0.06 in controls to 1.02 ± 0.15 in preHD and 1.18 ± 0.12 in HD [F(2,49) = 16.27, *p* < 0.0001; Cohen’s d = − 1.93], remaining significant after false discovery rate (FDR) correction. The putamen showed a parallel but more moderate increase [F(2,49) = 3.18, *p* = 0.050; d = − 0.93], whereas the caudate nucleus showed reduced DVR across disease stages [F(2,49) = 4.55, *p* = 0.015; d = 1.04], consistent with advanced neurodegeneration and loss of measurable tracer-accessible substrate. This finding should be interpreted cautiously because the caudate is particularly vulnerable to atrophy-related partial-volume effects and loss of tracer-accessible tissue.

**Fig. 2:**
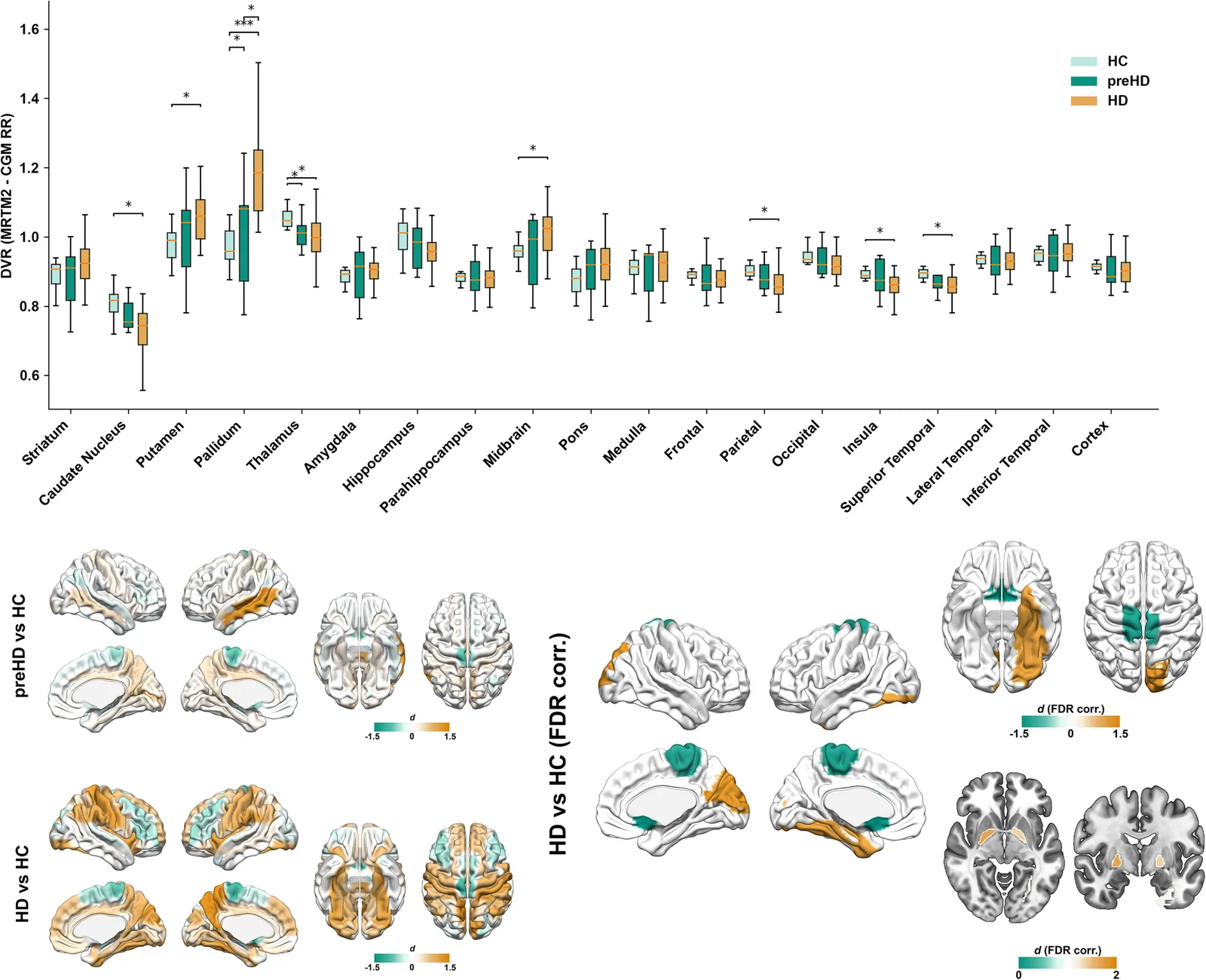
Group differences in [¹⁸F]PI-2620 tau-sensitive PET signal assessed with dynamic DVR. Group-level differences in [¹⁸F]PI-2620 PET signal quantified using DVR. Upper panels show ROI-based comparisons across HC, preHD, and manifest HD. Lower panels display whole-brain cortical and subcortical maps of effect sizes (Cohen’s d) for contrasts relative to controls. Warm colours indicate higher DVR and cool colours lower DVR in mutation carriers compared with controls. The figure highlights a predominantly subcortical pattern of tau-sensitive PET abnormality, with the largest and most spatially coherent effects localised to the globus pallidus and weaker, heterogeneous cortical involvement

Whole-brain analyses based on the full AAL parcellation supported this subcortical hierarchy (Fig. 2), revealing a bilaterally symmetric pallidal signal with the largest effect sizes observed across the brain. Putaminal involvement was present but weaker, while caudate effects were comparatively limited and spatially fragmented.

Cortical DVR differences were smaller but anatomically coherent, predominantly involving posterior associative regions. ROI analyses indicated modest reductions within parietal and superior temporal cortices, with similar patterns observed in fine-grained AAL analyses, particularly within transverse temporal, superior parietal, precuneus, and superior occipital regions. Complete region-wise results across the full AAL parcellation are provided in the Supplementary Results (S7) and Supplementary Table 1.

### ROI-based tau-sensitive PET positivity across disease stages

**Fig. 3:**
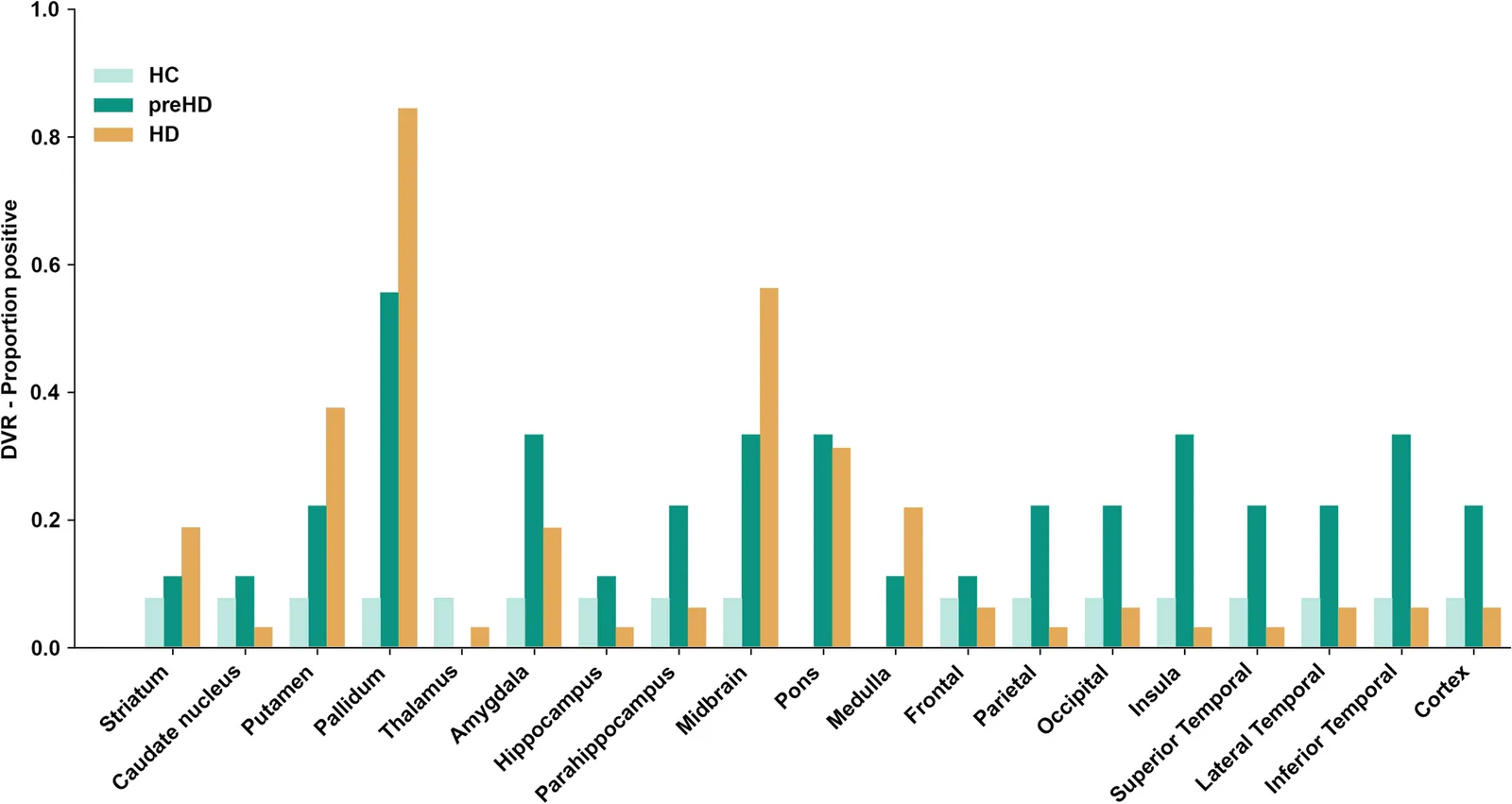
ROI-based tau-sensitive PET positivity across groups. Bar plots showing the proportion of participants classified as tau-sensitive PET positive (z ≥ 1.5 relative to healthy controls) for DVR. Bars represent positivity rates in HC, preHD, and manifest HD; only ROIs meeting predefined robustness criteria are shown. Pallidal positivity shows a monotonic stage-related increase across groups, whereas the caudate nucleus and thalamus show comparatively low positivity. Several limbic and cortical regions show non-monotonic cross-sectional profiles, with higher positivity in premanifest than manifest disease, indicating region- and stage-related heterogeneity

ROI-based positivity analyses revealed clear regional and cross-sectional stage-related differences in tau-sensitive PET abnormalities across the HD spectrum (Fig. 3). In HC, positivity rates were uniformly low across regions, generally ranging between 5 and 8% and rarely exceeding 10%, confirming the stringency of the selected threshold and the minimal false-positive burden.

In preHD, positivity increased in a spatially structured manner dominated by basal ganglia involvement. The globus pallidus showed the earliest and most prominent abnormalities, with approximately 45–55% of premanifest individuals exceeding the positivity threshold. Additional increases were observed in the putamen and midbrain, where approximately one-third of participants showed positive signal. In contrast, the caudate nucleus and thalamus remained largely spared at this stage, with positivity rates typically below 10–12%.

In manifest HD, subcortical positivity profiles diverged across nuclei. Pallidal positivity increased further and affected most participants (approximately 75–85%), consistent with a robust and predominantly monotonic stage-related increase in tau-sensitive PET positivity. Putaminal and midbrain positivity also increased modestly, reaching approximately 35–55%. In contrast, caudate and thalamic positivity remained comparatively low in manifest disease, rarely exceeding ~ 15%.

Several limbic regions exhibited a qualitatively different pattern suggestive of non-monotonic behaviour. Amygdalar positivity increased in preHD (approximately 30–45%) but declined in manifest HD to approximately 15–25%, yielding an intermediate-stage peak. Similar but less pronounced profiles were observed in hippocampal and parahippocampal regions.

Cortical regions showed lower and more heterogeneous positivity rates than subcortical structures. In preHD, modest positivity was observed in orbitofrontal, cingulate, insular, and selected posterior associative regions (around ~ 20%). In manifest HD, cortical positivity did not increase proportionally and often declined relative to preHD, with most regions remaining below ~ 10–12%. Complete region-wise positivity results across the full AAL parcellation are provided in the Supplementary Results (S8) and Supplementary Table 2.

### CAP-dependent probability of ROI-level tau-PET positivity

**Fig. 4:**
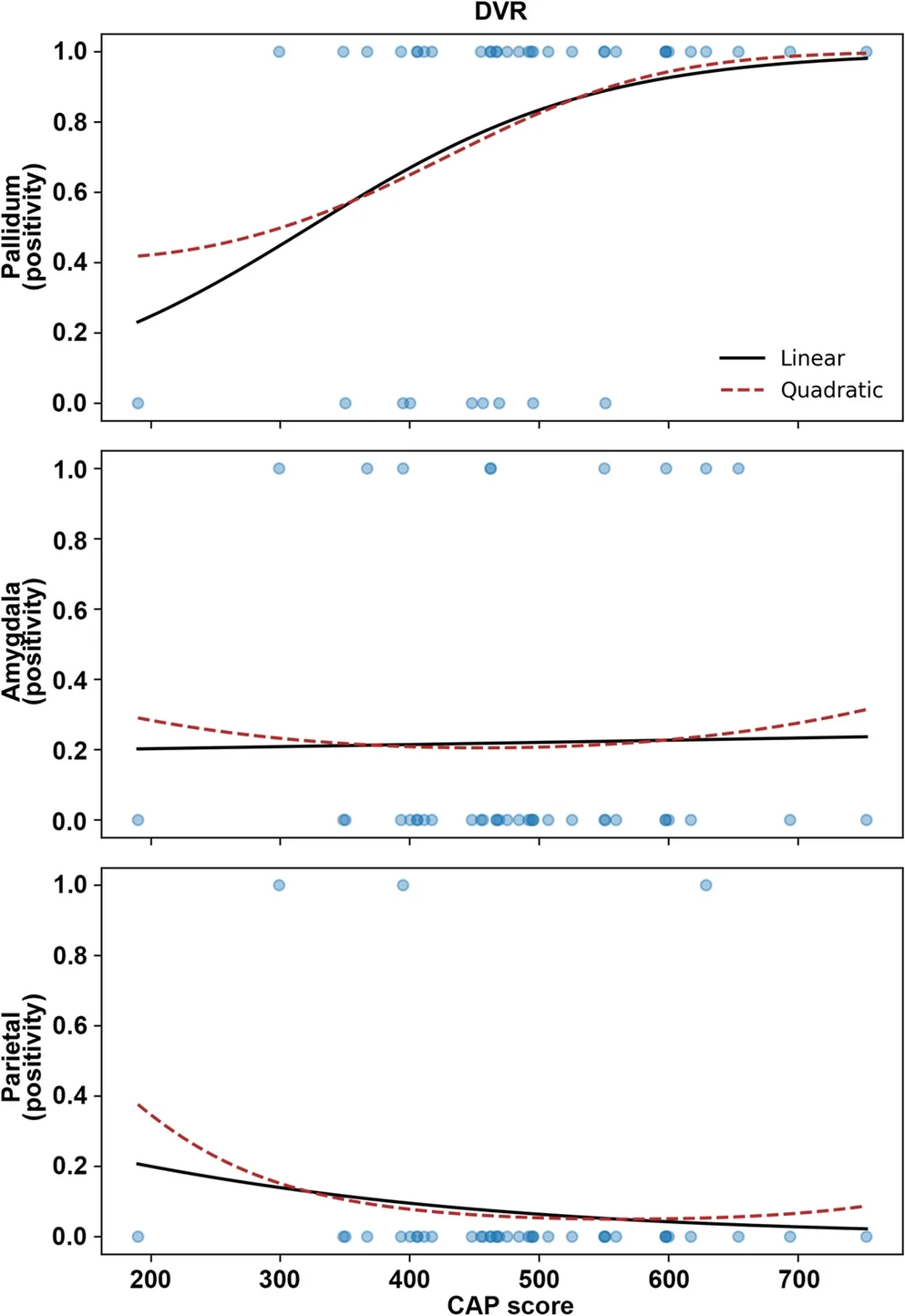
CAP-associated probability of regional tau-sensitive PET positivity. Individual-level tau-sensitive PET positivity (z ≥ 1.5 relative to controls) plotted as a function of CAP score for selected subcortical, limbic, and cortical regions. Points represent individual mutation carriers, with jitter applied to binary outcomes for visualisation. Solid lines show predicted probabilities from age- and sex-adjusted logistic regression models with a linear CAP term; dashed lines indicate models including a quadratic CAP term. Pallidal positivity shows a monotonic CAP-associated pattern for DVR, whereas limbic and cortical regions show sparse and heterogeneous positivity with limited evidence for CAP-dependent transitions

CAP-dependent modelling focused on three representative regions reflecting distinct anatomical compartments and positivity profiles (Fig. 4): the globus pallidus (subcortical; monotonic profile), the amygdala (limbic; profile suggestive of non-monotonic behaviour), and the parietal cortex (associative cortex; low-frequency profile). In mutation carriers, ROI-level tau-PET positivity was modelled as a binary outcome using age- and sex-adjusted logistic regression with CAP as the predictor. Both linear and exploratory quadratic CAP terms were evaluated, with likelihood ratio tests used to compare model fit (see Supplementary Methods S5).

The pallidum showed the clearest CAP-dependent relationship. Positivity increased from 55.6% in preHD to 84.4% in manifest HD, and higher CAP was associated with greater odds of pallidal tau-PET positivity (OR per + 10 CAP = 1.10, 95% CI 1.00–1.22; *p* = 0.059). Inclusion of a quadratic CAP term did not improve model fit (*p* = 0.339), supporting a predominantly monotonic CAP-related increase.

In contrast, limbic and cortical regions showed weaker and more heterogeneous CAP dependence. Amygdalar positivity was descriptively higher in premanifest than manifest carriers (33.3% vs. 18.8%), consistent with a profile suggestive of non-monotonic behaviour, but neither linear nor quadratic CAP terms reached significance (all *p* > 0.20). Parietal cortex positivity was infrequent and attenuated in manifest disease (22.2% vs. 3.1%), with no detectable CAP effects (all *p* > 0.70). Analyses using CAG repeat length showed a similar spatial pattern, with associations confined to pallidal regions (Supplementary Material S9).

### Stage-related heterogeneity of tau-PET–clinical associations

**Fig. 5:**
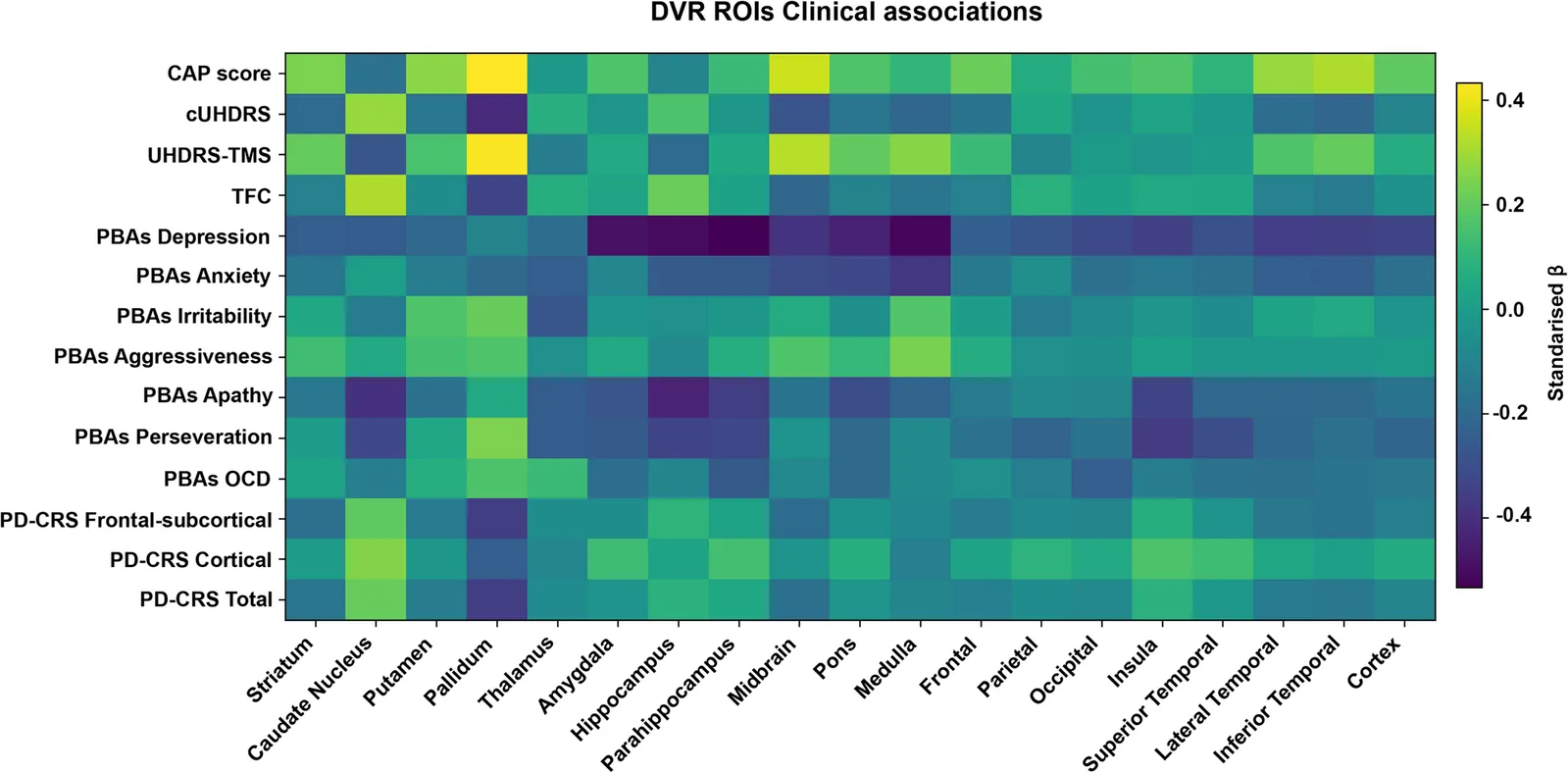
Regional tau-sensitive PET–clinical associations across disease domains. Heatmaps showing age- and sex-adjusted associations between regional [¹⁸F]PI-2620 DVR and clinical measures in mutation carriers. Colours represent standardised regression coefficients (β), with warm colours indicating positive and cool colours inverse associations. Subcortical regions, particularly the pallidum, show associations aligned with genetic and motor disease burden, whereas limbic and brainstem regions show prominent inverse associations with psychiatric symptom severity

Associations between regional tau-PET signal and clinical measures showed marked regional and domain-specific patterns (Fig. 5). Relationships with genetic and global disease burden were relatively focal and primarily confined to subcortical regions. Higher CAP scores were positively associated with pallidal DVR (β = +0.43, *p* = 0.002, q = 0.045), with an additional nominal association in the midbrain (β = +0.36, *p* = 0.024). Motor severity (UHDRS-TMS) showed similar nominal positive associations with pallidal and midbrain DVR, whereas global disease severity (cUHDRS) showed a nominal inverse association with pallidal DVR. Cognitive performance showed limited associations, with only nominal inverse relationships between pallidal DVR and PD-CRS scores.

In contrast, psychiatric symptoms accounted for the most spatially extensive associations. Depressive symptom severity was associated with lower DVR across multiple limbic and brainstem regions, with FDR-surviving inverse associations in the hippocampus, parahippocampus, amygdala, medulla, pons, and midbrain (β range − 0.39 to − 0.53, all q < 0.05). Apathy showed a similar but more restricted pattern, with an FDR-surviving inverse association in the hippocampus (β = −0.44, *p* = 0.002, q = 0.043).

**Fig. 6:**
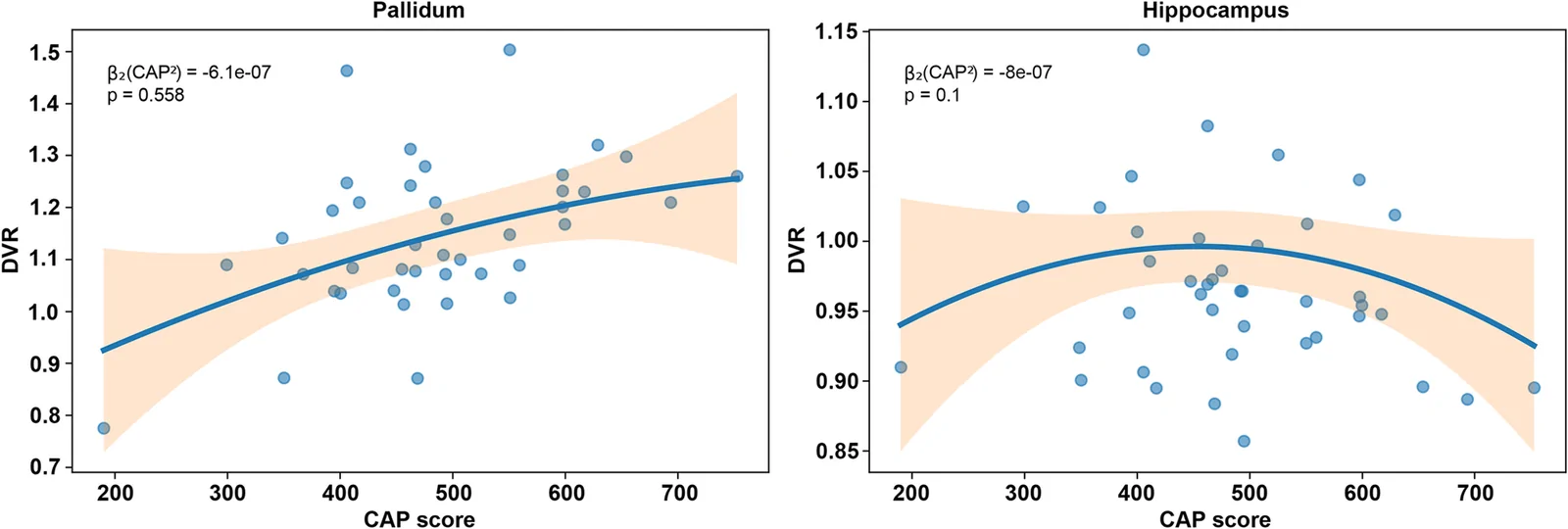
Cross-sectional CAP-associated tau-sensitive PET profiles using raw DVR values. Raw regional [¹⁸F]PI-2620 DVR values plotted as a function of CAP score in mutation carriers for a subcortical target region (pallidum) and a limbic region (hippocampus). Solid curves represent fitted values from age- and sex-adjusted models including linear and quadratic CAP terms, with shaded 95% confidence intervals. Pallidal DVR shows a monotonic CAP-associated increase, whereas hippocampal DVR shows a tendency towards attenuation at higher CAP levels, with wide confidence intervals reflecting substantial interindividual variability. The figure illustrates region-specific cross-sectional CAP-associated PET patterns and should not be interpreted as demonstrating within-subject trajectories

To determine whether these inverse associations reflected stage-dependent effects, additional interaction and non-linear models incorporating CAP terms were performed (see Supplementary Methods S6). These analyses indicated that tau-PET–clinical relationships in limbic regions varied as a function of cross-sectional disease stage. In the hippocampus, depressive symptom × CAP interaction effects suggested stronger associations at lower and intermediate CAP levels, with attenuation at higher CAP. In contrast, subcortical regions such as the pallidum showed stable associations with disease burden and no evidence of CAP-dependent modulation (Fig. 6).

### Discriminative performance of tau-PET

**Fig. 7:**
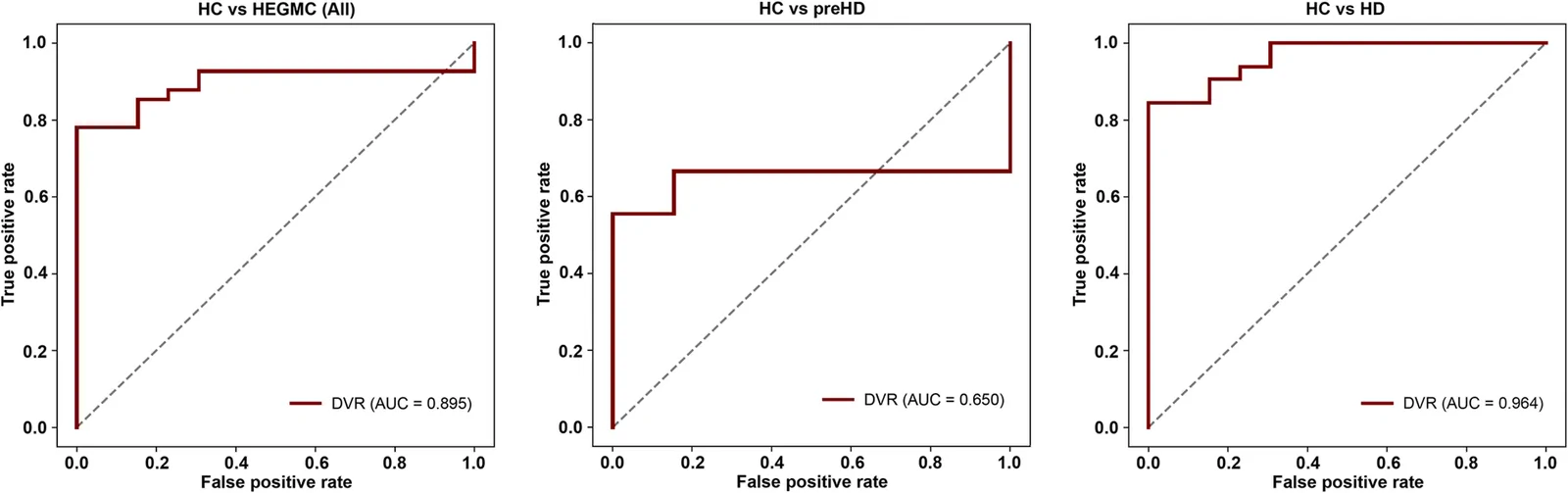
Discriminative performance of pallidal [¹⁸F]PI-2620 DVR across groups. Receiver operating characteristic (ROC) curves illustrating the group-level discriminative performance of pallidal [¹⁸F]PI-2620 DVR for distinguishing HC from mutation carriers, preHD, and manifest HD. Pallidal DVR shows high discriminative accuracy for distinguishing HC from manifest HD, whereas discrimination between HC and preHD carriers is limited

When distinguishing HC from all gene-expansion carriers, pallidal tau-PET DVR showed high discriminative accuracy. Pallidal DVR yielded an area under the ROC curve (AUC) of 0.895 (95% CI 0.80–0.97), with an optimal threshold around 1.07, achieving 78% sensitivity and 100% specificity. The AUC significantly exceeded chance level (DeLong test, *p* < 0.001), indicating that pallidal [¹⁸F]PI-2620 DVR provides group-level separation between healthy individuals and mutation carriers (Fig. 7). When analyses were restricted to discrimination between HC and preHD, performance was substantially reduced. Pallidal DVR achieved an AUC of 0.65 (95% CI 0.47–0.81), with discriminative performance not significantly different from chance (*p* > 0.10), indicating limited sensitivity of pallidal tau-PET to detect very early disease stages at the individual level. In contrast, discrimination between HC and manifest HD was excellent. Pallidal DVR reached an AUC of 0.964 (95% CI 0.91–1.00), achieving 84% sensitivity and 100% specificity. DeLong tests confirmed that the AUC was significantly above chance (*p* < 0.0001), indicating high group-level separability between controls and clinically manifest disease.

## Discussion

This study provides an in vivo characterisation of tau-sensitive [¹⁸F]PI-2620 PET signal in HD using dynamic kinetic modelling and complementary analytical approaches [43–45, 47, 48, 64]. Our findings reveal a highly stereotyped subcortical pattern dominated by pallidal and putaminal increases, accompanied by caudate DVR reductions and more modest thalamic and midbrain involvement. In parallel, we identify a posterior cortical signature involving Heschl’s gyrus, superior parietal cortex, precuneus, and superior occipital regions. Positivity analyses revealed a clear subcortical-to-cortical gradient, with early pallidal abnormalities already present in a subset of premanifest carriers and near-saturation of subcortical positivity in manifest disease. Tau-PET clinical associations were regionally heterogeneous: subcortical regions were linked to genetic and disease burden, whereas limbic and associative regions were primarily associated with psychiatric symptoms. Stage-related modelling further indicated that this heterogeneity partly reflects disease-stage modulation, linking non-monotonic positivity profiles with changes in tau-PET clinical relationships across CAP levels. Together, these findings indicate that tau-sensitive PET signal in HD is spatially ordered, biologically relevant, and cross-sectionally modulated across disease stages. Because the present study is cross-sectional, these stage-related patterns should be interpreted as inferred and hypothesis-generating rather than definitive within-subject trajectories.

The biological interpretation of [¹⁸F]PI-2620 signal in HD requires a balanced framework. [¹⁸F]PI-2620 is a second-generation tau PET tracer with extensive validation in Alzheimer’s disease and several primary tauopathy contexts, including evidence of high-affinity tau binding, favourable selectivity over amyloid-β, low non-specific white-matter binding, and in vivo retention in 4R tauopathy target regions [45, 46, 49, 64]. Therefore, a spatially structured and clinically associated [¹⁸F]PI-2620 signal should not be dismissed a priori as biologically meaningless or automatically off-target. At the same time, HD-specific autoradiographic validation is not yet available, and the precise molecular substrate of tracer retention in HD remains unknown. The term “tau-sensitive” is therefore used deliberately: the observed signal may reflect low-level or non-classical tau binding, 4R-enriched tau conformations, altered tracer kinetics in neurodegenerated tissue, tissue-composition effects, or off-target/non-specific components, particularly in subcortical regions [51, 52].

The most robust observation was the subcortical signature centred on the pallidum and putamen. Pallidal DVR showed the largest effect sizes across the brain and convergent evidence across continuous signal analyses, positivity metrics, CAP modelling, and ROC-based discrimination. While group contrasts often showed premanifest means between controls and manifest HD, positivity analyses revealed that many premanifest carriers already exceeded control-derived abnormality thresholds in the pallidum, highlighting early focal abnormalities not captured by group averages. CAP-dependent modelling supported a predominantly monotonic subcortical pattern. In the pallidum, the probability of tau-PET positivity increased with CAP, and quadratic terms did not improve model fit. This pattern aligns with established models of HD neurobiology in which basal ganglia circuits, particularly those projecting through the pallidum, represent early and severely affected nodes [1, 2, 8, 13]. This is particularly important for the globus pallidus, a region with high iron content, dense fibre architecture, complex myelin composition and marked vulnerability to HD-related neurodegenerative and glial changes. These features raise the possibility of altered tracer kinetics or non-specific components. However, several observations argue against interpreting the pallidal signal as a purely random or generic off-target artefact: it was bilaterally coherent, showed the largest effect size across the brain, emerged in positivity analyses, increased with CAP, related to motor/genetic burden, and showed high discrimination between controls and manifest HD. Thus, the pallidal finding is best interpreted as a robust tau-sensitive PET abnormality with unresolved molecular specificity [3, 8, 13].

The caudate nucleus illustrates the need to interpret tau-sensitive PET signal in the context of neurodegeneration and PET quantification constraints. Despite its prominent involvement in HD, caudate DVR was reduced in manifest disease. This finding is particularly vulnerable to partial-volume effects, because the caudate undergoes marked atrophy and tissue loss in HD. Although grey-matter-constrained ROIs were used to reduce CSF contamination and atrophy-related spill-out, this approach does not constitute formal partial-volume correction. No post-reconstruction PVC was applied, and PSF modelling was not enabled during image reconstruction. Therefore, reduced caudate DVR should not be interpreted as pathological sparing or absence of tau-related biology, but rather as a likely combination of tissue loss, loss of tracer-accessible substrate, spill-out effects, reference-region dependence and kinetic modelling constraints. At the same time, the overall basal ganglia pattern was not consistent with a simple global atrophy artefact: pallidal DVR increased markedly, putaminal DVR increased moderately, and caudate DVR decreased. Moreover, caudate positivity remained low even in premanifest carriers, where severe caudate atrophy is less likely to fully account for the absence of signal. Future studies incorporating formal PVC, regional volumetry and longitudinal structural-PET modelling will be required to resolve the contribution of atrophy to caudate quantification [65–67].

Cortical tau-PET abnormalities were smaller than subcortical changes but showed a reproducible posterior associative pattern involving transverse temporal, parietal, and occipital cortices, consistent with regions implicated in cognitive trajectories and cortical thinning in HD [4, 6–8]. Positivity profiling and stage-aware modelling suggested that several limbic and associative regions do not follow a simple monotonic cross-sectional stage-related profile. Regions such as the amygdala and hippocampus showed higher proportions of positive cases in premanifest than manifest HD, suggesting clustering of detectable tau-PET abnormality at intermediate disease burden. Continuous DVR and CAP analyses provided only modest statistical support for non-linearity, likely reflecting interindividual variability and limited event counts. Nevertheless, convergence across positivity, continuous signal, and clinical association analyses supports a conservative interpretation of intermediate-stage predominance rather than uniform accumulation. Given the cross-sectional design and limited representation of early stages, these findings should be regarded as hypothesis-generating regarding within-subject trajectories. Longitudinal imaging will be required to determine whether these cross-sectional profiles correspond to true within-subject temporal evolution.

Lower cortical DVR values observed in manifest HD warrant cautious interpretation. DVR estimates depend on reference region stability, and increased tracer retention in cerebellar grey matter could inflate the reference signal and produce apparent cortical reductions. In addition, advanced neuronal and synaptic loss may reduce tracer-accessible binding sites despite ongoing pathology. These mechanisms may contribute to lower cortical DVR values in later disease stages and highlight the importance of evaluating optimal reference region strategies for tau-PET quantification in HD [68–70].

Tau-PET clinical associations were strongly region- and domain-specific. Subcortical regions, particularly the pallidum, were associated with disease burden (CAP and motor severity), whereas limbic and brainstem regions showed inverse associations with psychiatric symptom severity in cross-sectional analyses. Stage-aware modelling indicated that these associations varied as a function of cross-sectional disease stage. In limbic regions such as the hippocampus, interaction and non-linear models suggested that psychiatric associations attenuate or change direction with increasing CAP, explaining why single linear models across all mutation carriers may yield net inverse relationships. Regions showing monotonic CAP-dependent increases in positivity also showed stable clinical associations, whereas regions with non-monotonic positivity profiles were precisely those in which clinical relationships varied across stages. These findings highlight the importance of explicitly modelling disease stage when interpreting cross-sectional tau-PET–clinical relationships in HD [68, 71, 72].

The present study prioritised dynamic kinetic modelling to provide a physiologically grounded characterisation of tau-sensitive [¹⁸F]PI-2620 signal in HD. Future work should evaluate simplified static quantification strategies and acquisition windows as complementary approaches for specific study designs. Evidence from other tauopathies suggests that early and late acquisition windows may differentially reflect binding to distinct tau isoforms, with potential relevance for the predominance of 4R tau species reported in HD [19, 20, 43–45, 47, 48]. Equally important is the systematic evaluation of optimal reference regions. Although cerebellar grey matter was used here in line with prior tau-PET studies, this choice should be considered pragmatic and literature-based rather than definitively validated for HD. Cerebellar involvement and cerebellar-network alterations are increasingly recognized across neurodegenerative diseases, and disease-related variation in cerebellar tracer uptake, tissue composition or atrophy could bias DVR estimates. Such bias would be particularly relevant for apparent cortical reductions and caudate findings. We did not replace cerebellar grey matter with an unvalidated alternative reference region, because alternative regions may introduce different and potentially larger biases in the absence of HD-specific validation. Future studies should evaluate HD-specific reference-region strategies, including alternative reference tissues and data-driven approaches [47, 69].

Several limitations should be acknowledged. First, the cross-sectional design precludes inference about within-subject temporal trajectories. Stage-related and CAP-associated profiles should therefore be interpreted as inferred and hypothesis-generating. Second, the modest sample size, particularly in premanifest carriers, limits power to detect subtle non-linear effects, CAP interactions and psychiatric associations. This is especially relevant for limbic and cortical regions with lower positivity rates and greater interindividual variability. Third, although MRI-based grey-matter-constrained ROIs were used to reduce CSF contamination and atrophy-related spill-out, no formal PVC was applied and PSF modelling was not enabled during reconstruction. Caudate reductions are therefore particularly vulnerable to partial-volume effects and should be interpreted cautiously. Fourth, cerebellar grey matter was used as the reference region in line with prior [¹⁸F]PI-2620 modelling work, but its stability in HD has not been specifically validated. Reference-region variability could influence DVR estimates, particularly cortical reductions and caudate findings. Fifth, no autoradiographic or post-mortem binding study has yet evaluated [¹⁸F]PI-2620 in HD tissue. The molecular substrate of the observed signal therefore remains unresolved, and the findings should be interpreted as tau-sensitive rather than tau-specific. Longitudinal studies combining tau-PET, structural MRI, fluid biomarkers, formal PVC and ultimately PET-to-pathology validation will be essential.

Despite these limitations, the present findings have important implications. Pallidal [¹⁸F]PI-2620 DVR showed excellent discrimination between controls and manifest HD, supporting its potential as a mechanistic and stratification biomarker in manifest or early manifest disease. However, discrimination in premanifest carriers was limited, and the present findings do not support tau-PET as a stand-alone screening or diagnostic biomarker. Instead, tau-sensitive PET should be considered within a broader multimodal biomarker framework that includes structural MRI, clinical staging, CAP/CAG-based measures, neurofilament light, mutant huntingtin, fluid tau markers and other molecular or neurodegeneration markers. More broadly, these findings help reconcile divergent literature on tau involvement in HD [19–24, 33, 35, 42, 73] by demonstrating that tau-related abnormalities are regionally and cross-sectionally stage-related rather than uniformly early or late.

In summary, this study provides a comprehensive in vivo characterisation of tau-sensitive [¹⁸F]PI-2620 PET signal in HD gene-expansion carriers, revealing a regionally ordered and cross-sectionally stage-related pattern that differs from age-related and Alzheimer-type distributions. By integrating kinetic modelling, positivity analyses, genetic burden, and stage-aware clinical modelling, the present work establishes a framework for interpreting tau-sensitive [¹⁸F]PI-2620 PET abnormalities in HD and highlights their relevance for understanding downstream disease mechanisms and therapeutic stratification. Future longitudinal, partial-volume-corrected and post-mortem validation studies will be required to define the temporal evolution and molecular specificity of this signal.

## Supplementary Information

Supplementary material is available at *Brain* online.

Supplementary Material 1

## Data Availability

The data that support the findings of this study are available from the corresponding author upon reasonable request. Public sharing is restricted due to confidentiality requirements as defined by the study protocol and ethical approvals.
