# Supplementary material for: Stage-dependent tau-PET signatures in Huntington’s disease revealed by [¹⁸F]PI-2620
Source: Eur J Nucl Med Mol Imaging. 2026 Jun 29;53(12):7376–90. doi: 10.1007/s00259-026-08003-0 (PMC13633314; doi:10.1007/s00259-026-08003-0)
Supplement: Supplementary file 1 [file 259_2026_8003_MOESM1_ESM.pdf]

Supplementary Material

Supplementary Methods S1. Clinical Assessment: Rationale for Cognitive and Psychiatric Measures

Global cognitive status was assessed using the Parkinson's Disease - Cognitive Rating Scale (PD-CRS), which provides a composite index of frontal-subcortical and posterior cortical functions. Although originally developed for Parkinson's disease[1], the PD-CRS has demonstrated high sensitivity to the global cognitive profile of HD and offers a broad evaluation of global cognition that aligns well with the multidomain deficits observed in this disorder[2-5]. In the context of the present study, the PD-CRS was selected as the principal cognitive measure because it captures overall cognitive status without overemphasizing single-process domains such as psychomotor speed or working memory, which are already incorporated into the composite Unified Huntington's Disease Rating Scale (cUHDRS)[6].

Neuropsychiatric symptoms were assessed with the short form of the Problem Behaviors Assessment (PBAs), yielding clinician-rated frequency and severity scores across ten behavioural domains. This instrument captures the spectrum of affective, motivational, and behavioural disturbances that characteristically emerge across the course of HD and provides a clinically interpretable index of psychiatric burden across disease stages.

Supplementary Methods S2:

MRI Acquisition Parameters and Processing Details

All participants underwent a high-resolution structural MRI scan acquired on a 3 Tesla Philips Ingeia system using a standardized T1-weighted MP-RAGE sequence. Acquisition parameters were as follows: repetition time (TR) = 8.16 ms, echo time (TE) = 3.74 ms, flip angle = 8°, acquisition matrix = 240 × 240, slice thickness = 1 mm, yielding a pixel spacing of 0.96 × 0.96 mm^2.

Structural MRI data were used for anatomical co-registration, spatial normalization, and tissue segmentation for PET quantification. Each PET image was rigidly registered to the individual T1-

weighted MRI. Nonlinear normalization to MNI space was then performed using the MRI-derived deformation fields. Gray matter tissue probability maps were calculated and intersected with the Automated Anatomical Labeling (AAL) atlas to define gray-matter–constrained regions of interest for PET analyses[7]. This grey-matter-constrained ROI approach was used to reduce cerebrospinal fluid contamination and atrophy-related spill-out effects, but it should not be considered equivalent to formal partial-volume correction.

PET acquisition and kinetic modelling

[^{18}F]PI-2620 PET data were acquired on a Philips Vereos digital PET/CT scanner (Philips Healthcare, Eindhoven, The Netherlands). Imaging was performed in list-mode and reconstructed using ordered-subset expectation maximisation (OSEM; 4 iterations, 16 subsets). Corrections were applied for attenuation, scatter, random coincidences, and dead time. Point-spread-function modelling was not enabled during reconstruction, and no post-reconstruction partial-volume correction was applied.

Dynamic PET acquisition began simultaneously with intravenous radiotracer injection and continued for 60 minutes. Following visual quality control, dynamic frames were corrected for head motion and co-registered to each participant's structural T1-weighted MRI. Spatial normalisation to MNI space was then performed using the MRI-derived transformations.

Tracer binding was quantified using distribution volume ratios (DVR) estimated with the multilinear reference tissue model (MRTM2). This approach has previously been validated for dynamic [^{18}F]PI-2620 imaging against arterial input function measurements[55].

Cerebellar grey matter (excluding the vermis and adjacent anterior lobe) was used as the reference region. Model parameters were set to $k2' = 0.22\ \text{min}^{-1}$. A starting time of $t^* = 0$ was used to improve voxel-wise model convergence in the present dataset compared with the original validation implementation ($t^* = 20$ min).

DVR maps generated from MRTM2 served as the primary outcome measure for all subsequent region-of-interest and voxel-wise analyses. DVR was interpreted as an operational reference-tissue kinetic outcome reflecting relative tracer distribution between target and reference tissue, rather than as a direct quantitative measure of fibrillar tau burden.

Supplementary Methods S3. Statistical Analysis

S3.1 Clinical and Demographic Characterisation

Descriptive analyses were undertaken to characterise the demographic, genetic, and clinical profile of the sample across healthy controls, premanifest mutation carriers, and clinically manifest HD participants. Continuous variables were summarised using means and standard deviations, and categorical variables using frequencies and percentages. Group differences were evaluated using general linear models or ANCOVA, adjusting for age and sex when appropriate. For ordinal or non-normally distributed outcomes, non-parametric tests were applied.

For continuous outcomes involving more than two groups, post-hoc pairwise comparisons were derived from the fitted models, yielding adjusted means and 95% confidence intervals. Effect sizes were calculated to quantify the magnitude of between-group differences. Correlations between clinical measures were explored using Pearson or Spearman coefficients, with age and sex included as covariates when indicated.

S3.2 Interpretation Framework for Multiregional PET Analyses

Given the multiregional and exploratory nature of the PET analyses, interpretation did not rely exclusively on nominal statistical significance. Instead, results were evaluated based on a convergent framework incorporating (i) effect sizes, (ii) anatomical coherence across neighbouring regions, and (iii) consistency between DVR-derived patterns. This approach was used to contextualise findings derived from both aggregated macro-regions and fine-grained AAL-defined regions and to support biologically meaningful interpretation of spatially distributed effects. The analytical hierarchy was defined to distinguish primary from complementary analyses. Primary analyses focused on group differences in ROI-based DVR measures, with emphasis on subcortical regions and the globus

pallidus. Positivity profiling, full AAL mapping, quadratic CAP models, clinical × CAP interaction models and ROC analyses were considered complementary or exploratory analyses intended to characterize regional heterogeneity and generate hypotheses for future studies.

Supplementary Methods S4. ROI-based tau-sensitive PET positivity: Robustness criteria

Regional tau-sensitive PET positivity was defined using DVR-derived z-scores calculated relative to the mean and standard deviation of the healthy control group. A priori, positivity was defined as $z \geq 1.5$, corresponding to tracer binding clearly exceeding the control distribution while maintaining a low false-positive rate.

For each region and disease stage, both the proportion and the absolute number of positive individuals were recorded. To ensure robustness and to limit the influence of isolated extreme values, regional positivity was considered meaningful only when at least two participants within a given group exceeded the predefined threshold.

Complete positivity results across the full set of AAL regions, for both DVR-based metrics, are provided in the Supplementary Results and Supplementary Tables.

Supplementary Methods S5. CAP-dependent modelling of tau-sensitive PET positivity

To examine whether the probability of regional tau-sensitive PET positivity varied systematically as a function of cumulative genetic disease burden, we modelled positivity status ($z \geq 1.5$ vs < 1.5) as a binary outcome using regression-based approaches restricted to mutation carriers.

As a primary approach, age- and sex-adjusted logistic regression models were implemented to assess monotonic CAP-dependent effects on the odds of tau-PET positivity:

$$\text{logit}\{P(\text{positive})\} = \alpha + \beta_1(\text{CAP}) + \beta_2(\text{Age}) + \beta_3(\text{Sex})$$

To evaluate whether selected regions exhibited evidence of non-monotonic, cross-sectional intermediate-stage peaks in positivity, complementary models incorporating a quadratic CAP term were fitted[8].

$$\text{logit}\{P(\text{positive})\} = \alpha + \beta_1(\text{CAP}) + \beta_2(\text{CAP}^2) + \text{covariates}$$

A significant negative quadratic coefficient ($\beta_2 < 0$) was interpreted as evidence for a non-linear relationship characterised by an intermediate-stage maximum in positivity.

To minimise post hoc selection and enhance interpretability, CAP–positivity modelling was restricted to a predefined subset of ROIs selected according to objective and biologically motivated criteria. ROIs were chosen to represent three anatomical compartments (subcortical nuclei, limbic structures, and associative cortex) and were required to show either (i) a monotonic increase in the proportion of positive cases across disease stages, or (ii) a non-monotonic pattern characterised by higher positivity in premanifest carriers than in manifest disease. Selection was based exclusively on descriptive positivity analyses and was independent of clinical association results.

For each selected ROI and PET metric, monotonic CAP effects were first evaluated using a linear CAP term, and potential non-linearity was assessed by adding a quadratic CAP term. Likelihood ratio tests were used to compare quadratic versus linear specifications. Effect sizes are reported as odds ratios (ORs) per +10 CAP points with 95% confidence intervals[9,10].

Supplementary Methods S6. Clinical-Anatomical Association Models

Associations between regional tau-PET signal and clinical measures were examined using age- and sex-adjusted linear regression models restricted to mutation carriers. Initial analyses were conducted in an exploratory framework to characterise the regional distribution, direction, and magnitude of associations across the brain.

Where exploratory analyses suggested heterogeneous or non-uniform association patterns, interaction models were specified to test whether associations varied as a function of cumulative disease burden:

$$\text{Tau-PET}_{ROI} = \beta_0 + \beta_1(\text{Clinical measure}) + \beta_2(\text{CAP}) + \beta_3(\text{Clinical measure} \times \text{CAP}) + \beta_4(\text{Age}) + \beta_5(\text{Sex}) + \varepsilon$$

To evaluate non-linear cross-sectional CAP-associated profiles of regional tau-sensitive PET signal, additional models incorporating a quadratic CAP term were fitted:

$$\text{Tau-PET}_{ROI} = \beta_0 + \beta_1(\text{CAP}) + \beta_2(\text{CAP}^2) + \beta_3(\text{Age}) + \beta_4(\text{Sex}) + \varepsilon$$

Evidence for non-linearity was assessed using likelihood ratio tests comparing quadratic and linear CAP models. A significant negative quadratic term was interpreted as indicating cross-sectional profiles characterised by early increases, intermediate plateaus, and attenuation at higher disease burden, rather than definitive within-subject trajectories[10-12.]

Supplementary Results (S7):

To ensure complete spatial coverage and analytical transparency, group-level comparisons of tau-sensitive PET signal were additionally performed across all regions defined by the AAL atlas, using the same analytical framework applied to anatomically aggregated macro-regions in the main manuscript.

For each AAL-defined region, raw DVR values were entered into age- and sex-adjusted general linear models, with HD-ISS group as the main factor. Pairwise contrasts were computed between controls and each mutation-carrier group. For each comparison, adjusted mean differences, twosided p values, false discovery rate (FDR)-corrected q values (Benjamini–Hochberg, $q = 0.05$), and Cohen's d effect sizes were derived.

To enhance readability and facilitate interpretation, Supplementary Table S1 reports only those AAL regions showing statistically significant group differences ($p < 0.05$ or FDR-corrected $q <$ 0.05).

Within subcortical structures, the most prominent DVR increases were consistently observed in the pallidal regions. Both left and right pallidum exhibited large effect sizes ($|d|$ approximately 1.82.0) and remained significant after FDR correction in the HC vs HD comparison, confirming a bilateral and anatomically symmetric effect. Putaminal regions showed more heterogeneous behaviour: the left

putamen demonstrated a significant DVR increase with moderate-to-large effect size that survived FDR correction, whereas the right putamen displayed a parallel but weaker gradient that did not meet corrected significance thresholds.

As in the main manuscript, these caudate reductions should be interpreted cautiously because caudate quantification is particularly vulnerable to atrophy-related partial-volume effects, tissue loss and loss of tracer-accessible substrate.This effect was more pronounced in the left caudate, which survived FDR correction, while the right caudate exhibited a similar but subthreshold pattern. No caudate AAL region showed significant differences between controls and preHD.

At the cortical level, significant DVR reductions were spatially restricted and anatomically coherent. The strongest and most consistent effects were observed in posterior and auditory– parietal regions. Bilateral reductions were detected in the transverse temporal (Heschl's) gyri, both hemispheres surviving FDR correction with large effect sizes. Additional reductions were observed in the superior parietal lobule and precuneus, with left-hemispheric parcels more frequently surviving FDR correction than their right-sided counterparts. A focal reduction was also evident in the right superior occipital gyrus, which remained significant after FDR correction, whereas the corresponding left region did not.

Across cortical AAL regions, DVR differences were driven almost exclusively by the transition from control to manifest disease, with no region showing a significant separation between controls and preHD.

Supplementary Table 1. Group-level differences across all AAL Regions

AAL region	Hemisphere	Metric	Comparison	Difference	CI95 low	CI95 high	p value	*d*	q value
Calcarine *	Left	DVR	HC vs HD	-0.027	-0.054	-0.001	0.044	-0.837	0.159
CaudateNucl ***	Left	DVR	HC vs HD	-0.104	-0.158	-0.049	0.000	-1.300	0.008
CaudateNucl *	Right	DVR	HC vs HD	-0.068	-0.129	-0.007	0.028	-0.833	0.130
Cerebellum8 *	Right	DVR	HC vs HD	0.020	0.003	0.037	0.021	0.880	0.111
Cingulum_Mid ***	Left	DVR	HC vs HD	-0.047	-0.077	-0.016	0.003	-1.186	0.038
Cingulum_Mid *	Right	DVR	HC vs HD	-0.042	-0.073	-0.011	0.009	-1.156	0.071
Cuneus *	Left	DVR	HC vs HD	-0.033	-0.065	-0.001	0.046	-0.842	0.159
Cuneus *	Right	DVR	HC vs HD	-0.034	-0.065	-0.002	0.035	-0.868	0.149
Heschl ***	Left	DVR	HC vs HD	-0.088	-0.119	-0.057	0.000	-1.971	0.000
Heschl ***	Right	DVR	HC vs HD	-0.082	-0.110	-0.055	0.000	-1.959	0.000
Insula *	Left	DVR	HC vs HD	-0.028	-0.056	-0.000	0.048	-0.839	0.163
Insula *	Right	DVR	HC vs HD	-0.028	-0.053	-0.003	0.027	-0.895	0.130
Midbrain *	-	DVR	HC vs HD	0.057	0.007	0.106	0.026	0.899	0.130
Occipital Sup *	Left	DVR	HC vs HD	-0.035	-0.068	-0.003	0.034	-0.954	0.149
Occipital Sup ***	Right	DVR	HC vs HD	-0.048	-0.079	-0.018	0.002	-1.325	0.032
Pallidum ***	Left	DVR	HC vs HD	0.189	0.118	0.260	0.000	1.889	0.000

Pallidum ***	Right	DVR	HC vs HD	0.212	0.135	0.289	0.000	1.922	0.000
Paracentral Lobule *	Right	DVR	HC vs HD	-0.064	-0.113	-0.014	0.012	-0.880	0.093
Parietal Inf *	Left	DVR	HC vs HD	-0.051	-0.093	-0.010	0.017	-1.003	0.106
Parietal Inf ***	Right	DVR	HC vs HD	-0.067	-0.109	-0.025	0.002	-1.267	0.032
Parietal Sup ***	Left	DVR	HC vs HD	-0.065	-0.104	-0.026	0.002	-1.362	0.026
Parietal Sup ***	Right	DVR	HC vs HD	-0.064	-0.106	-0.022	0.004	-1.251	0.041
Postcentral *	Left	DVR	HC vs HD	-0.042	-0.074	-0.009	0.014	-1.013	0.095
Postcentral *	Right	DVR	HC vs HD	-0.036	-0.068	-0.004	0.028	-1.010	0.130
Precentral *	Right	DVR	HC vs HD	-0.035	-0.068	-0.001	0.041	-0.859	0.153
Precuneus *	Left	DVR	HC vs HD	-0.041	-0.070	-0.012	0.007	-1.138	0.067
Precuneus *	Right	DVR	HC vs HD	-0.036	-0.066	-0.007	0.017	-1.081	0.106
Putamen *	Left	DVR	HC vs HD	0.085	0.023	0.146	0.008	1.007	0.068
Putamen *	Right	DVR	HC vs HD	0.058	0.002	0.113	0.041	0.806	0.153
Temporal Pole Sup *	Left	DVR	HC vs HD	-0.038	-0.074	-0.002	0.040	-0.702	0.153
Temporal Sup *	Left	DVR	HC vs HD	-0.042	-0.071	-0.013	0.006	-1.130	0.057
Thalamus *	Left	DVR	HC vs HD	-0.044	-0.080	-0.008	0.018	-0.908	0.106
Thalamus *	Right	DVR	HC vs HD	-0.048	-0.088	-0.008	0.019	-0.906	0.109
Cerebellum_Crus2 *	Left	DVR	HC vs preHD	-0.024	-0.045	-0.004	0.021	-1.165	0.835
Heschl *	Left	DVR	HC vs preHD	-0.051	-0.092	-0.011	0.014	-1.007	0.819
Heschl *	Right	DVR	HC vs preHD	-0.051	-0.087	-0.016	0.005	-0.768	0.646

For ease of interpretation, Supplementary Table S1 including only regions showing statistically significant group differences (* $p < 0.05$ or *** FDR-corrected $q < 0.05$)

Supplementary Results (S8):

Regional tau-sensitive PET positivity was assessed across the full AAL parcellation using a z-score–based approach relative to healthy controls. For each AAL-defined region, z-DVR values were calculated using the mean and standard deviation of the control group as reference. A priori positivity thresholds were defined as $z \geq 1.5$.

Using these thresholds, regional positivity rates were quantified separately for preHD and HD groups. For each region and group, both the proportion of positive individuals and the absolute number of positive cases were recorded. Consistent with the main analyses, regional positivity was considered meaningful only when at least two individuals within a group exceeded the predefined threshold.

Complete DVR-based positivity data across all AAL regions are provided in Supplementary Table 2. To enhance readability and to focus on regions exhibiting meaningful deviations from the control distribution, Supplementary Table S2 reports only those AAL regions showing detectable tau-sensitive PET positivity ($z \geq 1.5$)

The positivity analysis across all AAL regions provided a complementary, threshold-based view of the group-level effects observed in the continuous analyses. Using an a priori threshold of $z \geq 1.5$ relative to

the control group, regional positivity was quantified separately for preHD and HD participants across the full AAL parcellation.

Using this definition, regional positivity was infrequent in preHD and spatially restricted, whereas HD participants showed broader and more consistent positivity across subcortical regions. Positivity was most consistently observed within pallidal and putaminal regions, paralleling the structures that exhibited the largest effect sizes in the group-level DVR analyses.

Caudate regions showed comparatively low positivity rates despite demonstrating DVR reductions in the continuous analyses, indicating a dissociation between threshold-based abnormality and group-level signal differences in these structures. At the cortical level, positivity was sparse and regionally selective, predominantly involving posterior temporal-parietal regions, including transverse temporal, superior parietal, and precuneus parcels.

Across regions, heterogeneous stage-related patterns were evident. Some AAL regions showed detectable positivity already in preHD that did not markedly increase in HD, whereas other regions exhibited minimal positivity in preHD followed by higher positivity rates in HD. This regional heterogeneity mirrors the non-uniform patterns observed in the continuous group-level analyses and highlights that tau-sensitive PET abnormalities are not uniformly expressed across regions or cross-sectional disease stages.

Across both DVR-based metrics, regions exhibiting higher positivity rates largely overlapped with those showing larger effect sizes in the group-level comparisons, supporting internal consistency between continuous signal differences and z-score–based measures of abnormality when considering the full AAL parcellation.

Supplementary Table S2:

AAL region	Hemisphere	Metric	HC (%)	preHD (%)	HD (%)
Amygdala	Left	DVR	7.7	33.3	21.9
Amygdala	Right	DVR	7.7	44.4	12.5
Angular	Left	DVR	7.7	22.2	6.2
Angular	Right	DVR	15.4	22.2	3.1
Calcarine	Left	DVR	7.7	11.1	0.0
Calcarine	Right	DVR	7.7	11.1	3.1
Caudate	Left	DVR	7.7	11.1	3.1
Caudate	Right	DVR	7.7	11.1	3.1
Cerebellum10	Left	DVR	7.7	22.2	6.2
Cerebellum10	Right	DVR	7.7	33.3	12.5
Cerebellum3	Left	DVR	7.7	11.1	18.8
Cerebellum3	Right	DVR	7.7	22.2	12.5
Cerebellum45	Left	DVR	7.7	11.1	15.6

Cerebellum45	Right	DVR	7.7	22.2	12.5
Cerebellum6	Left	DVR	7.7	11.1	9.4
Cerebellum6	Right	DVR	7.7	33.3	6.2
Cerebellum7	Left	DVR	0.0	0.0	3.1
Cerebellum7	Right	DVR	7.7	0.0	6.2
Cerebellum8	Left	DVR	7.7	0.0	9.4
Cerebellum8	Right	DVR	7.7	22.2	50.0
Cerebellum9	Left	DVR	7.7	0.0	12.5
Cerebellum9	Right	DVR	7.7	0.0	9.4
Cerebellum_Crus1	Left	DVR	0.0	0.0	12.5
Cerebellum_Crus1	Right	DVR	0.0	22.2	9.4
Cerebellum_Crus2	Left	DVR	7.7	0.0	3.1
Cerebellum_Crus2	Right	DVR	7.7	0.0	0.0
Cingulum Ant	Left	DVR	7.7	33.3	6.2
Cingulum Ant	Right	DVR	7.7	33.3	9.4
Cingulum Mid	Left	DVR	7.7	33.3	3.1
Cingulum Mid	Right	DVR	7.7	33.3	3.1
Cingulum Post	Left	DVR	7.7	11.1	9.4
Cingulum Post	Right	DVR	0.0	33.3	6.2
Cuneus	Left	DVR	7.7	22.2	6.2
Cuneus	Right	DVR	7.7	22.2	6.2
Frontal Inf Oper	Left	DVR	7.7	11.1	3.1
Frontal Inf Oper	Right	DVR	7.7	22.2	6.2
Frontal Inf Orb	Left	DVR	7.7	22.2	28.1
Frontal Inf Orb	Right	DVR	7.7	33.3	34.4
Frontal Inf Tri	Left	DVR	7.7	22.2	6.2
Frontal Inf Tri	Right	DVR	7.7	11.1	12.5
Frontal Med Orb	Left	DVR	7.7	33.3	15.6
Frontal Med Orb	Right	DVR	7.7	33.3	12.5
Frontal Mid	Left	DVR	7.7	11.1	3.1
Frontal Mid	Right	DVR	7.7	11.1	6.2
Frontal Mid Orb	Left	DVR	7.7	22.2	28.1
Frontal Mid Orb	Right	DVR	7.7	33.3	37.5
Frontal Sup	Left	DVR	7.7	11.1	3.1
Frontal Sup	Right	DVR	7.7	11.1	6.2
Frontal Sup Medial	Left	DVR	7.7	11.1	12.5
Frontal Sup Medial	Right	DVR	7.7	11.1	6.2
Frontal Sup Orb	Left	DVR	7.7	33.3	28.1
Frontal Sup Orb	Right	DVR	7.7	33.3	28.1
Fusiform	Left	DVR	7.7	22.2	15.6
Fusiform	Right	DVR	7.7	22.2	15.6
Heschl	Left	DVR	7.7	0.0	0.0
Heschl	Right	DVR	7.7	0.0	0.0
Hippocampus	Left	DVR	0.0	0.0	3.1
Hippocampus	Right	DVR	7.7	11.1	3.1
Insula	Left	DVR	7.7	22.2	3.1
Insula	Right	DVR	7.7	33.3	3.1
Lingual	Left	DVR	7.7	11.1	6.2
Lingual	Right	DVR	7.7	11.1	6.2
Medulla	-	DVR	0.0	11.1	21.9
Midbrain	-	DVR	7.7	33.3	56.2

Occipital_Inf	Left	DVR	7.7	11.1	9.4
Occipital Inf	Right	DVR	7.7	22.2	25.0
Occipital Mid	Left	DVR	7.7	22.2	3.1
Occipital Mid	Right	DVR	7.7	22.2	3.1
Occipital Sup	Left	DVR	7.7	22.2	6.2
Occipital Sup	Right	DVR	7.7	22.2	0.0
Olfactory	Left	DVR	7.7	44.4	12.5
Olfactory	Right	DVR	7.7	11.1	6.2
Pallidum	Left	DVR	7.7	44.4	75.0
Pallidum	Right	DVR	0.0	55.6	84.4
Paracentral Lobule	Left	DVR	7.7	11.1	6.2
Paracentral Lobule	Right	DVR	7.7	22.2	9.4
Parahippocampus	Left	DVR	7.7	11.1	6.2
Parahippocampus	Right	DVR	7.7	22.2	6.2
Parietal Inf	Left	DVR	7.7	22.2	3.1
Parietal Inf	Right	DVR	7.7	11.1	3.1
Parietal Sup	Left	DVR	7.7	22.2	3.1
Parietal Sup	Right	DVR	0.0	11.1	3.1
Pons		DVR	0.0	33.3	31.2
Postcentral	Left	DVR	7.7	11.1	3.1
Postcentral	Right	DVR	7.7	11.1	3.1
Precentral	Left	DVR	7.7	11.1	3.1
Precentral	Right	DVR	7.7	11.1	3.1
Precuneus	Left	DVR	7.7	22.2	3.1
Precuneus	Right	DVR	7.7	22.2	9.4
Putamen	Left	DVR	7.7	33.3	43.8
Putamen	Right	DVR	7.7	22.2	34.4
Rectus	Left	DVR	7.7	11.1	15.6
Rectus	Right	DVR	7.7	11.1	12.5
Rolandic Oper	Left	DVR	7.7	22.2	6.2
Rolandic Oper	Right	DVR	7.7	22.2	9.4
Supp Motor Area	Left	DVR	7.7	22.2	6.2
Supp Motor Area	Right	DVR	7.7	22.2	6.2
Supra Marginal	Left	DVR	7.7	22.2	3.1
Supra Marginal	Right	DVR	7.7	11.1	9.4
Temporal Inf	Left	DVR	7.7	11.1	9.4
Temporal Inf	Right	DVR	7.7	22.2	15.6
Temporal Mid	Left	DVR	7.7	22.2	3.1
Temporal Mid	Right	DVR	15.4	11.1	6.2
Temporal Pole Mid	Left	DVR	7.7	22.2	12.5
Temporal Pole Mid	Right	DVR	7.7	22.2	21.9
Temporal Pole Sup	Left	DVR	15.4	22.2	9.4
Temporal Pole Sup	Right	DVR	7.7	11.1	6.2
Temporal Sup	Left	DVR	7.7	11.1	3.1
Temporal Sup	Right	DVR	7.7	22.2	6.2
Thalamus	Left	DVR	7.7	0.0	3.1
Thalamus	Right	DVR	0.0	11.1	9.4

Supplementary Results (S9): CAG repeat length and regional tau-sensitive PET signal

To determine whether mutation size independently influenced tau-sensitive PET signal, associations between CAG repeat length and regional DVR were examined in mutation carriers using age- and sex-adjusted linear regression models. CAG repeat length showed a focal association pattern with regional tau-PET signal, largely confined to subcortical regions. Higher CAG repeat length was robustly associated with increased pallidal DVR ($\beta = 0.75$–0.80, $p < 10^{-4}$), accounting, together with age and sex, for a substantial proportion of variance in pallidal binding. This association survived false discovery rate correction and was consistently observed across left and right pallidal subregions.

More moderate positive associations were also observed within the broader striatal complex, including the striatum and putamen, with effect sizes smaller than those observed in the pallidum and limited spatial extent. In contrast, caudate and thalamic DVR showed no significant associations with CAG repeat length once age was accounted for.

Outside the basal ganglia, no meaningful CAG dependence was observed. Cortical regions, including those showing clear group-level DVR differences, exhibited small, non-significant coefficients, indicating that cortical tau-PET signal is not directly modulated by mutation size alone. A similar absence of association was observed in limbic regions, suggesting that CAG repeat length does not account for the heterogeneous tau-PET clinical relationships identified in earlier analyses.

References:

1. Pagonabarraga J, Kulisevsky J, Llebaria G, Garcia-Sanchez C, Pascual-Sedano B, Gironell A. Parkinson's disease-cognitive rating scale: a new cognitive scale specific for Parkinson's disease. *Mov Disord*. May 15 2008;23(7):998-1005. doi:10.1002/mds.22007

2. Martinez-Horta S, Horta-Barba A, Perez-Perez J*, et al*. Utility of the Parkinson's diseaseCognitive Rating Scale for the screening of global cognitive status in Huntington's disease. *J Neurol*.
May 2020;267(5):1527-1535. doi:10.1007/s00415-020-09730-6

3. Martinez-Horta S, Puig-Davi A, Sampedro F*, et al*. Neural and biomarker correlates of the Parkinson's Disease-Cognitive Rating Scale in Huntington's disease. *Neuroimage Rep*. Mar 2026;6(1):100311. doi:10.1016/j.ynirp.2025.100311

4. Martinez-Horta S, Sampedro F, Horta-Barba A*, et al*. Structural brain correlates of dementia in Huntington's disease. *Neuroimage Clin*. 2020;28:102415. doi:10.1016/j.nicl.2020.102415

5. Horta-Barba A, Martinez-Horta S, Perez-Perez J*, et al*. Measuring cognitive impairment and monitoring cognitive decline in Huntington's disease: a comparison of assessment instruments. *J Neurol*.
Nov 2023;270(11):5408-5417. doi:10.1007/s00415-023-11804-0

6. Schobel SA, Palermo G, Auinger P*, et al*. Motor, cognitive, and functional declines contribute to a single progressive factor in early HD. *Neurology*. Dec 12 2017;89(24):2495-2502.
doi:10.1212/WNL.0000000000004743

7. Tzourio-Mazoyer N, Landeau B, Papathanassiou D*, et al*. Automated anatomical labeling of activations in SPM using a macroscopic anatomical parcellation of the MNI MRI single-subject brain.
Neuroimage. Jan 2002;15(1):273-89. doi:10.1006/nimg.2001.0978

8. Applied multiple regression/correlation analysis for the behavioral sciences, 3rd ed. Mahwah, NJ, US.

9. Kriegeskorte N, Simmons WK, Bellgowan PS, Baker CI. Circular analysis in systems neuroscience: the dangers of double dipping. *Nat Neurosci*. May 2009;12(5):535-40.
doi:10.1038/nn.2303

10. Harrell FE, Jr. *Regression Modeling Strategies: With Applications to Linear Models, Logistic and Ordinal Regression, and Survival Analysis*. Springer Series in Statistics. Springer International Publishing; 2015.

11. Poldrack RA, Fletcher PC, Henson RN, Worsley KJ, Brett M, Nichols TE. Guidelines for reporting an fMRI study. *Neuroimage*. Apr 1 2008;40(2):409-414.
doi:10.1016/j.neuroimage.2007.11.048

12. Multiple regression: Testing and interpreting interactions. Thousand Oaks, CA, US.
